# Scale‐Specific Viscoelastic Characterization of Hydrogels: Integrated AFM and Finite Element Modeling

**DOI:** 10.1002/smll.202507835

**Published:** 2025-12-03

**Authors:** Nicole Fertala, Klemens Uhlmann, Evgeny Grigoryev, Prannoy Seth, Jens Friedrichs, Julian Thiele, Carsten Werner, Daniel Balzani

**Affiliations:** ^1^ Leibniz Institute of Polymer Research Dresden Division Polymer Biomaterials Science Max Bergmann Center of Biomaterials Hohe Straße 6 01069 Dresden Germany; ^2^ Ruhr University Bochum Chair of Continuum Mechanics Universitätsstraße 150 44801 Bochum Germany; ^3^ Leibniz Institute of Polymer Research Dresden Institute of Physical Chemistry and Polymer Physics Hohe Strasse 6 01069 Dresden Germany; ^4^ Otto von Guericke University Magdeburg Institute of Chemistry Universitätsplatz 2 39106 Magdeburg Germany; ^5^ Dresden University of Technology Center of Regenerative Therapies Dresden and Cluster of Excellence Physics of Life Fetscherstraße 105 01307 Dresden Germany

**Keywords:** atomic force microscopy, finite element modeling, interpenetrating polymer networks, scale‐dependent mechanics, viscoelastic hydrogels

## Abstract

Viscoelastic hydrogels mimic the dynamic mechanical properties of native extracellular matrices, making them essential for biomedical applications. However, characterizing their scale‐dependent mechanical properties remains challenging, despite their critical influence on cell‐material interactions and biomaterial performance. Here, an integrated experimental‐computational approach is presented to quantify and model the viscoelastic behavior of interpenetrating polymer network hydrogels across micro‐ and macro‐scales. Atomic force microscopy‐based stress relaxation tests revealed that microgels exhibit rapid, localized relaxation, while macroscopic bulk gels displayed prolonged relaxation dominated by poroelastic effects. Finite element simulations accurately replicated experimental conditions, enabling the extraction of key parameters: fully relaxed elastic modulus, relaxation modulus, and relaxation time constant. A novel analytical model is further developed to predict viscoelastic parameters from experimental data with minimal error (<6%), significantly streamlining characterization. The findings highlight the necessity of scale‐specific mechanical analysis and provide a robust platform for designing biomaterials with tailored viscoelasticity for tissue engineering and regenerative medicine.

## Graphical abstract

An integrated multiscale approach reveals distinct viscoelastic responses of hydrogels across length scales. Combining AFM‐based nanoindentation, finite element modeling, and a novel analytical framework, this study enables accurate, scale‐specific mechanical characterization to guide the design of advanced biomaterials.

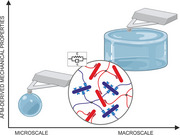

## 1 Introduction

Hydrogels have transformed biomedical engineering by mimicking the dynamic, water‐rich environment of native extracellular matrices (ECM) through tunable, three‐dimensional polymer networks derived from natural or synthetic sources.^[^ ^1^, ^2^, ^3^ ^]^ These materials replicate the porous architecture and biochemical signaling of tissues, enabling physiologically relevant models that surpass traditional 2D cultures. As such, hydrogels are integral to advances in targeted drug delivery,^[^ ^4^, ^5^, ^6^, ^7^ ^]^ cell encapsulation,^[^ ^8^, ^9^ ^]^ tissue engineering,^[^ ^10^, ^11^, ^12^, ^13^ ^]^ biosensing, and mechanobiology.^[^ ^14^, ^15^, ^16^, ^17^, ^18^ ^]^

A key advantage of hydrogels is their dual tunability in both chemical composition and structural scale. Bulk hydrogels typically serve as robust scaffolds for tissue regeneration, promoting cell adhesion, proliferation, and differentiation, while enabling advanced applications such as 3D bioprinting and organ‐on‐a‐chip platforms.^[^ ^19^, ^20^, ^21^, ^22^, ^23^, ^24^ ^]^ In contrast, microscale hydrogel particles, known as microgels (ranging from 0.1 to 100 µm in diameter), excel in cell encapsulation, controlled drug release, and biosensing,^[^ ^25^, ^26^ ^]^ with recent studies demonstrating their ability to guide endothelial morphogenesis through electrostatic microenvironment modulation.^[^ ^27^ ^]^

Despite their growing utility, microgel mechanics are often extrapolated from bulk gel measurements, assuming that identical formulations yield consistent behavior across scales. However, significant discrepancies exist between micro‐ and macroscale properties of the same material.^[^ ^28^, ^29^ ^]^ These differences critically impact biological performance, microgel stiffness and stress relaxation directly affecting cell proliferation, differentiation, and mechanotransduction.^[^ ^30^, ^31^, ^32^, ^33^, ^34^ ^]^ Inaccurate characterization thus risks compromising biomedical outcomes.

Recent advances in hydrogel technology focus on viscoelastic systems that more accurately recapitulate the time‐dependent mechanical behavior of living tissues.^[^ ^35^, ^36^, ^37^, ^38^, ^39^ ^]^ Unlike purely elastic materials, viscoelastic hydrogels resist immediate deformation and dissipate energy under load, mimicking ECM remodeling during physiological processes such as wound healing.^[^ ^40^, ^41^, ^42^ ^]^ However, characterizing these dynamics – particularly at microscales – remains challenging. While bulk rheometry captures macroscale viscoelasticity, probing microgels demands specialized techniques such as atomic force microscopy (AFM)–based nanoindentation.^[^ ^43^, ^44^ ^]^ Standard stress relaxation tests under constant strain are used to characterize the complex, time‐dependent responses of soft materials.^[^ ^45^, ^46^, ^47^ ^]^ However, the models commonly employed often oversimplify the system, leading to inaccurate estimates of relaxation time and creep compliance, or they have so far been established only for bulk hydrogels.

**Figure 1:**
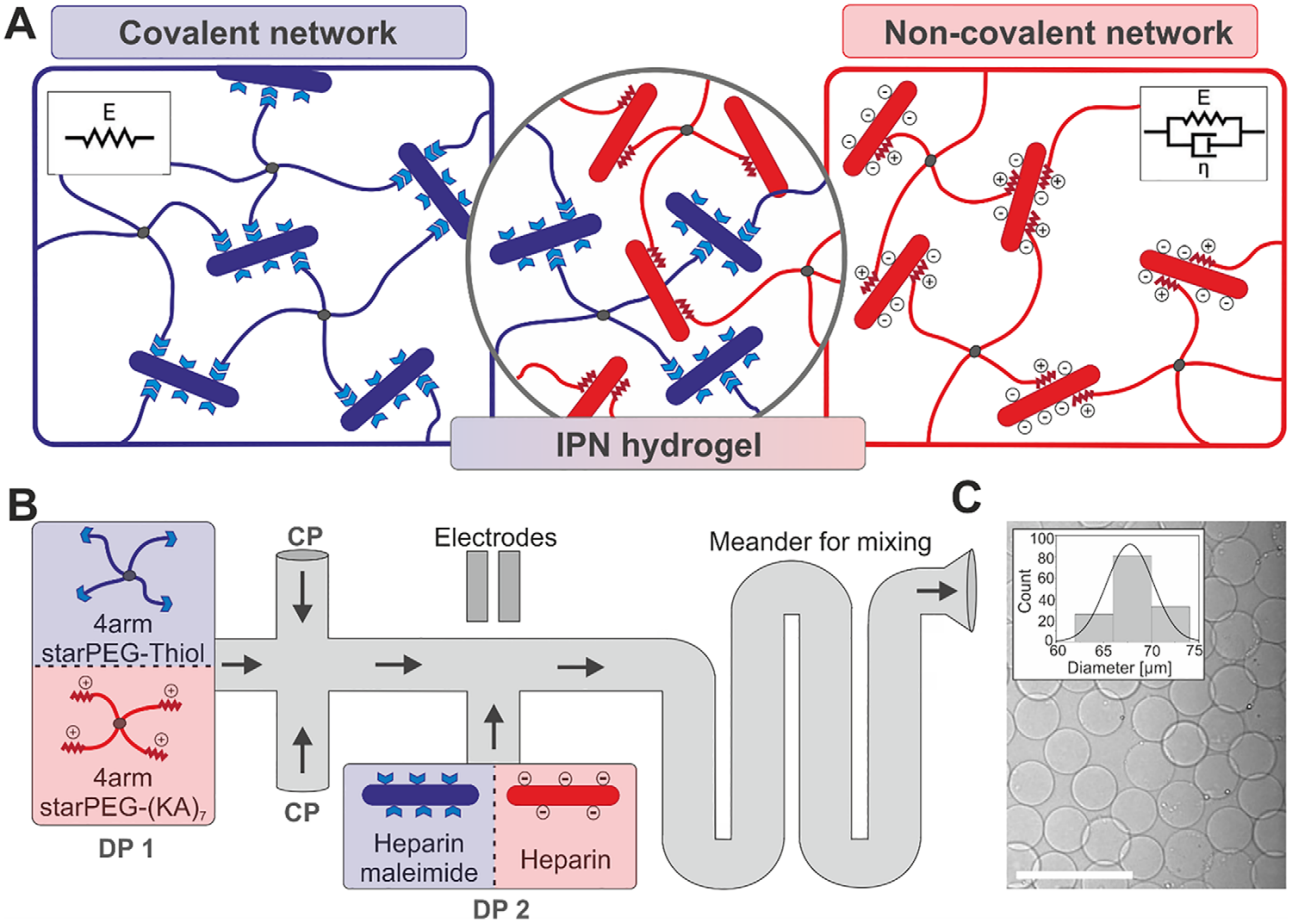
Composition and fabrication of viscoelastic IPN microgels. A) Schematic representation of the viscoelastic interpenetrating polymer network (IPN) hydrogel platform. The hydrogel consists of two networks: a covalent network (blue) formed by a Michael‐type addition reaction between thiolated starPEG and maleimide‐functionalized heparin provides robust elastic properties. Concurrently, a physically crosslinked network (red) formed via reversible electrostatic interactions between sulfated heparin and starPEG conjugated with the heparin‐binding peptide (KA)_7_ imparts viscous properties and controlled stress relaxation.^[^ ^48^ ^]^ B) Microfluidic fabrication of IPN microgels.^[^ ^55^ ^]^ A microfluidic device equipped with integrated electrodes enables rapid and precise mixing of the continuous phase (CP) of fluorinated oil to which a surfactant is added for droplet stabilization and the precursor solutions. Droplets containing PEG‐based precursors (dispersed phase 1 – DP 1) and heparin‐based precursors (dispersed phase 2 – DP 2) are generated separately, merged at a T‐junction through electric field‐induced droplet fusion, and uniformly mixed within a downstream meandering channel to form monodisperse microgels. Arrows indicate fluid flow directions. **C)** Brightfield microscopy image of the resulting viscoelastic IPN microgels. The inset shows the size distribution of the microgels (mean = 67.8 ± 2.4 µm, n = 140). Scale bar: 250 µm.

To address these challenges, we employ previously established viscoelastic interpenetrating polymer network (IPN) hydrogels^[^ ^48^ ^]^ as model systems to investigate correlations in viscoelastic parameters across multiple scales. These hydrogels feature a dual‐network architecture: (1) a covalent network formed through Michael‐type addition between thiolated four‐armed poly ethylene glycol (starPEG) and maleimide‐functionalized sulfated glycosaminoglycan heparin, providing structural integrity and elastic behavior; and (2) a physical network arising from reversible electrostatic interactions between heparin and heparin‐binding peptides conjugated to starPEG, imparting a time‐dependent viscous response (**Figure** **1**A).^[^ ^49^, ^50^ ^]^ This design allows precise control over both stiffness and stress relaxation behavior, making these IPN hydrogels ideal for exploring scale‐dependent viscoelastic phenomena.

By integrating AFM‐based stress relaxation tests with finite element (FE) simulations, we dissected scale‐dependent viscoelasticity, revealing that bulk gels exhibit prolonged poroelastic relaxation – where water migration through an extensive pore network contributes to a viscous response,^[^ ^51^, ^52^, ^53^, ^54^ ^]^ while microgels display rapid, localized relaxation. These scale‐dependent differences underscore that inferring microgel properties from bulk gel measurements can lead to substantial discrepancies in key parameters such as relaxation time and creep compliance.

We further developed an analytical model to predict the viscoelastic properties of microgels with error margins below 6%, streamlining characterization. This approach directly correlates AFM measurements with optimized material parameters, offering unprecedented accuracy in capturing both elastic and viscous contributions to hydrogel behavior. Ultimately, our approach bridges micro‐macro mechanical discrepancies, offering critical insights for biomaterial design.

## 2 Results and Discussion

### 2.1 Formation of Viscoelastic Microgels and Bulk Hydrogels

Viscoelastic microgels and bulk hydrogels were fabricated using an established cell‐instructive interpenetrating polymer network (IPN) hydrogel platform (Figure 1A).^[^ ^48^ ^]^ This system combines two distinct networks: (1) a covalent network formed through Michael‐type addition between thiolated star‐shaped polyethylene glycol (starPEG) and maleimide‐functionalized heparin that provides elastic properties; and (2) a physical network of reversible electrostatic interactions between heparin‐binding peptides ((KA)_7_) conjugated to starPEG and negatively charged heparin sulfate groups, imparting viscous properties and enabling controlled stress relaxation. By adjusting the ratio of these two networks, the viscoelastic profile of the hydrogel can be precisely tuned to mimic natural tissue mechanics.

For the first time, monodisperse microgels were fabricated from this IPN system using microfluidic techniques with integrated electrodes, enabling rapid and controlled mixing of hydrogel precursors via emulsion droplet coalescence (Figure 1B).^[^ ^55^ ^]^ Droplets of the PEG‐based precursor solution (DP 1) were generated at a flow‐focusing junction using a fluorinated oil continuous phase (CP) stabilized by surfactant. These droplets were then guided to merge with heparin‐based precursor droplets (DP 2) at a T‐junction, where an applied electric field (50 kV) triggered rapid coalescence by destabilizing the water‐oil interface. Subsequent mixing in a meandering channel facilitated uniform gelation, producing highly monodisperse microgels (diameter = 67.8 ± 2.4 µm, Figure 1C).

### 2.2 Mechanical Characterization and FE Simulations Reveal Scale‐Dependent Behavior

**Figure 2:**
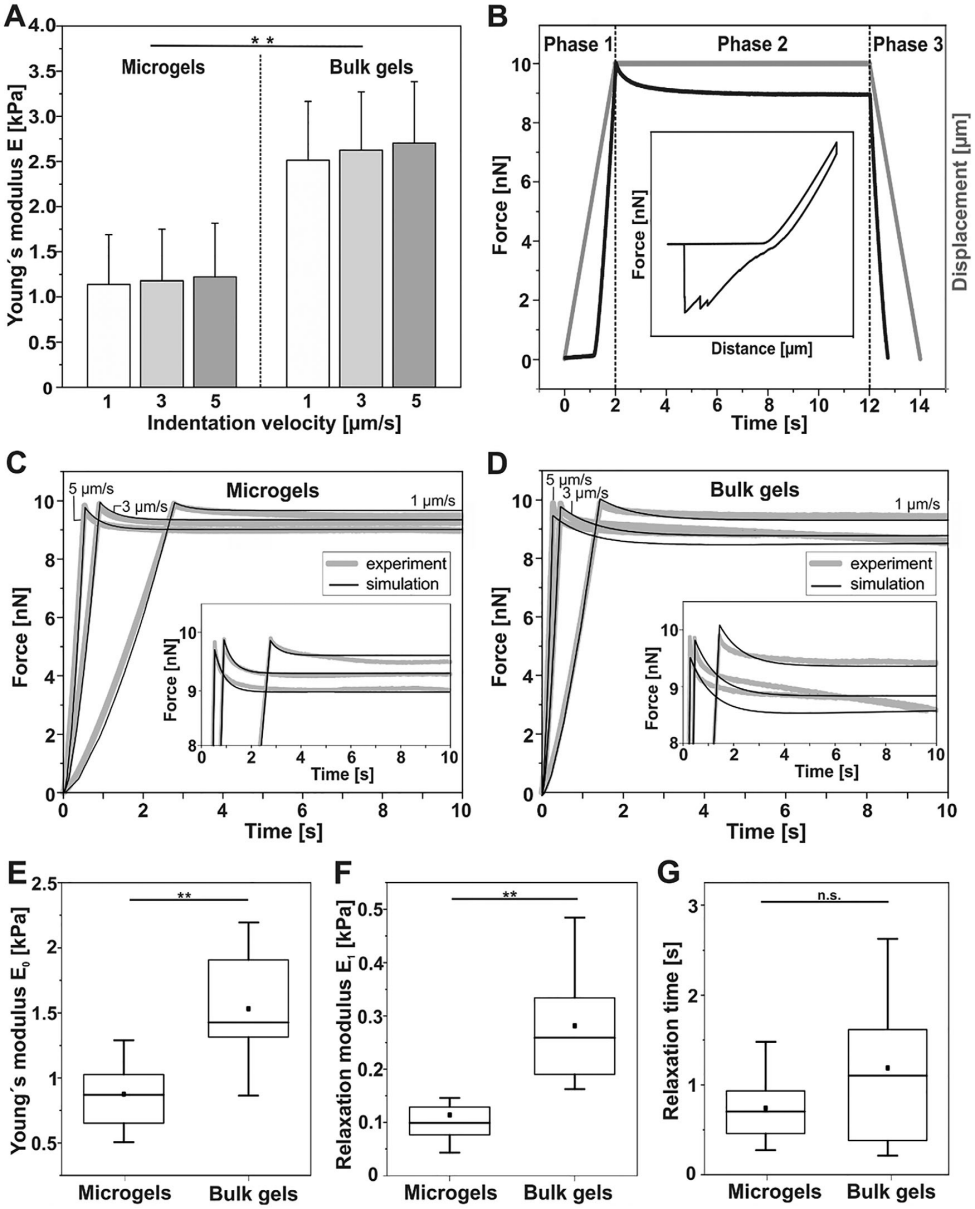
Comparative mechanical characterization of viscoelastic microgels and bulk hydrogels. A) AFM‐based nanoindentation analysis comparing elasticity (Young's modulus, *E*) of microgels and bulk gels at varying indentation velocities. Data are presented as mean ± SD (n = 10). B) Schematic illustration of the three phase AFM‐based stress relaxation protocol: Phase 1 (Indentation): The cantilever approaches and indents the sample at constant velocity until a predefined contact force (10 nN) is reached. Phase 2 (Stress Relaxation): The cantilever position is held constant (for 10 s) at maximum displacement, to monitor force decay due to material relaxation. Phase 3 (Retraction): The cantilever is withdrawn from the sample surface. The graph shows representative cantilever height (grey) and contact force (black) profiles over time, with inset illustrating the force–height relationship. C, D) Comparison of experimental (grey) and simulated (black) force relaxation curves for microgels (C) and bulk gels (D) during the 10‐second holding period of phase 2. While the model accurately captures the rapid relaxation of microgels, it systematically fails to describe the prolonged, non‐equilibrating relaxation of bulk gels, a characteristic signature of poroelastic effects. Insets provide enlarged views of phase 2 for both microgels and bulk gels, highlighting the detailed relaxation dynamics at the probe–sample interface. E–G) FE‐derived viscoelastic parameters. Box plots show 25th/75th percentiles (boxes), means (squares), medians (lines), and standard deviation (whiskers) (n = 10), **P <0.01 (Wilcoxon–Mann–Whitney test).

To investigate the scale‐dependent mechanical properties of the IPN hydrogel, we conducted AFM‐based nanoindentation experiments on both microgels and bulk hydrogels prepared from identical precursor compositions. In these experiments, a probe of defined geometry indents the sample while the cantilever deflection measures the applied force. Fitting the resulting force–indentation curves to the Hertz model yielded the Young's modulus (*E*). Our results revealed that microgels are significantly more compliant than bulk gels, and both formats stiffen with increasing indentation velocity, indicating pronounced time‐dependent viscoelastic behavior (**Figure** **2**A).

To further characterize the viscoelastic properties, we performed stress relaxation tests at multiple indentation velocities, following a three‐phase protocol (Figure 2B). In the first phase, the AFM cantilever approached the sample surface at constant velocity until reaching a predetermined indentation force, inducing instantaneous elastic deformation. Continuous recording of cantilever deflection during this phase provided force–indentation data directly correlated to the material's instantaneous elastic modulus. These initial measurements serve as the baseline for understanding the load‐bearing capabilities of the sample. In the second phase, the cantilever position was held constant at maximum indentation for 10 s, during which the initial stress decays as the polymer network rearranges. This phase yields key viscoelastic parameters – relaxation modulus (*E_1_*) and time constant (*τ*) – while the residual stress isolates the purely elastic component (**inset,** Figure 2B). In the third phase, the cantilever was retracted from the sample surface. However, this phase was omitted from quantitative analysis due to adhesion artifacts that obscure intrinsic material behavior.

Both microgels (Figure 2C) and bulk gels (Figure 2D) demonstrated distinct velocity‐dependent relaxation profiles characteristic of viscoelastic materials. To quantitatively extract viscoelastic parameters from these curves, FE simulations replicating the AFM experiments were performed (see Experimental Section). The simulated stress relaxation curves (black lines, Figure 2C, D) closely matched experimental data across different indentation velocities, validating the measurement approach and confirming the observed velocity dependence.

However, bulk gels displayed significant deviations during the relaxation phase (phase 2), particularly at higher indentation velocities, characterized by prolonged and inconsistent relaxation patterns (Figure 2C, D).

This discrepancy likely arises from two competing relaxation mechanisms: (1) intrinsic viscoelasticity arising from polymer network rearrangements, and (2) poroelastic effects caused by fluid migration through the gel's porous structure during sustained deformation.^[^ ^56^, ^57^, ^58^ ^]^ The intrinsic dual‐network architecture of the IPN hydrogel contributes to these behaviors: the covalent network, formed via a Michael‐type addition between thiolated starPEG and maleimide‐functionalized heparin, provides a permanent elastic scaffold that defines the equilibrium stiffness and constrains solvent mobility, while the physically crosslinked network, stabilized by reversible electrostatic interactions between sulfated heparin and starPEG conjugated with the heparin‐binding peptide (KA)_7_, introduces dynamic crosslinks that enable time‐dependent stress relaxation and viscous dissipation. Together, these permanent and reversible interactions not only dictate the viscoelastic relaxation characteristics but also modulate the poroelastic response by transiently affecting pore connectivity and fluid transport within the network.

A central finding of our work is that the measured mechanical response of the IPN hydrogel is scale‐dependent. It is crucial to clarify that this does not mean the material properties themselves change; both microgels and bulk gels are fabricated from the same intrinsically viscoelastic material. Instead, the observed differences arise because the measured response of the bulk gel is a convolution of its intrinsic viscoelasticity and a dominant, scale‐dependent poroelastic effect. The evidence for this is twofold. First, bulk gels exhibit prolonged, non‐converging relaxation characteristic of poroelastic fluid flow (Figure 2D). Second, and as a direct consequence, our purely viscoelastic FE model systematically fails to capture this long‐term relaxation in bulk gels, while it accurately describes the rapid, localized relaxation of the microgels (Figure 2C). This differential performance of the model is not a contradiction; it is the key finding that confirms that while the microgel response is dominated by intrinsic viscoelasticity, an additional poroelastic mechanism governs the bulk gel response.

This distinction is physically well‐founded and stems from the strong influence of dimensional constraints on poroelasticity. The extensive pore network of macroscopic bulk gels allows for significant, long‐range solvent migration, leading to prolonged relaxation. Conversely, the small size of microgels restricts fluid flow, making their response dominated by the intrinsic viscoelasticity of the polymer network. This intrinsic viscoelasticity is a designed feature of our dual‐network IPN hydrogel, where the physically crosslinked network of reversible electrostatic interactions introduces dynamic crosslinks that enable time‐dependent stress relaxation.

These differences may be further modulated by fabrication‐induced structural variations, as active mixing for bulk gels may introduce more heterogeneous pore structures than the diffusion‐driven assembly used for microgels. Taken together, the combination of size‐dependent effects, velocity‐dependent relaxation, and the systematic deviation from a viscoelastic‐only model confirms that poroelastic mechanisms play a significant role in the relaxation of bulk gels, while their contribution is minimal in microgels.

Direct comparison of the FE‐derived mechanical parameters revealed significant scale‐dependent differences between microgels and bulk hydrogels, despite identical hydrogel composition. Specifically, microgels showed significantly lower fully relaxed Young's moduli (*E_0_*) compared to bulk gels (Figure 2E), along with substantially reduced relaxation moduli (*E_1_*; Figure 2F) and shorter relaxation times (*τ*; Figure 2G). These findings demonstrate that hydrogel mechanical properties cannot be simply extrapolated across length scales, highlighting the critical importance of scale‐specific mechanical characterization for accurate biomaterial design and application.

### 2.3 Characterization of Material Parameters Using Predefined Equations

**Figure 3:**
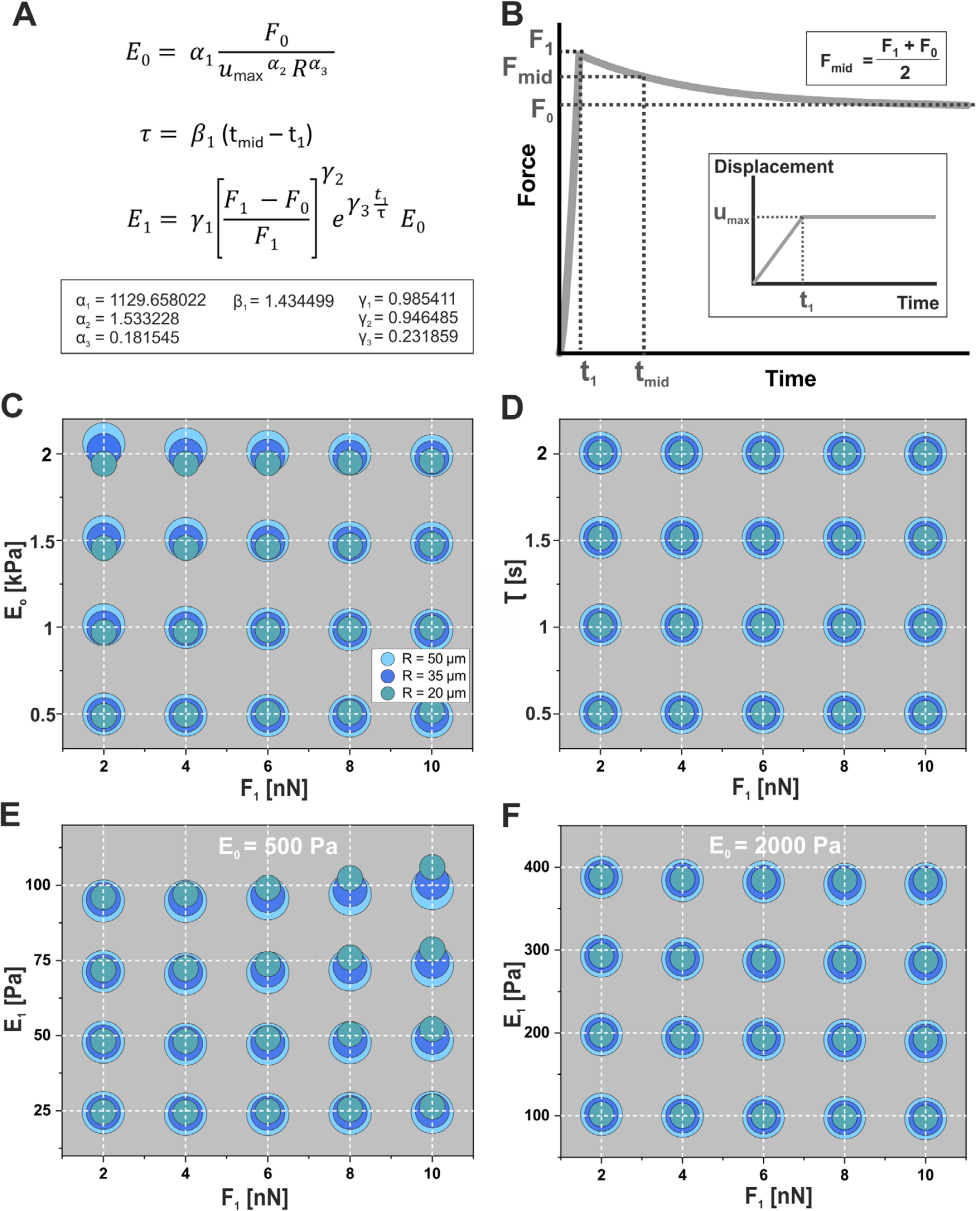
Determination of viscoelastic material parameters and associated errors: A) Predefined equations enable fast estimation of key viscoelastic parameters – fully relaxed Young's modulus (*E_0_*), relaxation modulus (*E_1_*), and relaxation time constant (*τ*) – directly from AFM‐based stress relaxation data. B) Representative force–time and indentation–time curves illustrating the experimental input parameters required for the calculations shown in (A). C–F) Error analysis comparing estimated parameter values (dots) to actual values (grid intersections) as functions of the maximum contact force (*F_1_*) and the microgel radius (*R*): (C) Maximum absolute errors for fully relaxed Young's modulus (*E_0_*). (D) Maximum absolute errors for relaxation time (*τ*), observed under conditions of highest relaxation (cantilever velocity *v* = 5 µm s^−1^, *E_0_* = 2000 Pa, *E_1_* = 400 Pa). (E, F) Maximum absolute errors for relaxation modulus (*E_1_*) at the lower (*E_0_* = 500 Pa, E) and upper (*E_0_* = 2000 Pa, F) limits of tested fully relaxed Young's moduli under conditions of lowest relaxation.

While the described optimization procedure accurately determines viscoelastic material parameters, it becomes inefficient when applied to large experimental datasets. To address this limitation, we developed a streamlined analytical approach based on a set of predefined equations that extract key viscoelastic parameters from just four experimental measurements (**Figure** **3**A). In addition to these four measurements, the microgel radius (*R*) and the maximum indentation force (*F_1_*, marking the transition between phases 1 and 2) are required. The governing equations are:

(1) $$E_{0}=\alpha_{1}\frac{F_{0}}{{u_{\max}}^{\alpha_{2}}R^{\alpha_{3}}}$$

(2) $$\tau =\beta_{1}\left(t_{mid}-t_{1}\right)$$

(3) $$E_{1}=\gamma_{1}{\left[\frac{F_{1}-F_{0}}{F_{1}}\right]}^{\gamma_{2}}{e^{\gamma_{3}}}^{\frac{t_{1}}{\tau }}E_{0}$$

where *F_1_* is the maximum contact force between cantilever and microgel, *F_0_* is the residual contact force at the end of phase 2, *t_1_* and *t_mid_* denote the start and midpoint times of phase 2 (with *F_mid_* = (*F_1_* + *F_0_*)/2), and *u_max_* is the maximum indentation depth during phase 2 (Figure 3B). The coefficients (α_1_ α_2_, α_3_, β_1_, γ_1_, γ_2_, γ_3_) were optimized across experimentally relevant ranges of microgel radii, maximum contact forces, cantilever velocities, and material parameters (*E_0_* *, E_1_/E_0_* and *τ*, see Experimental Section).

Using these optimized coefficients, the predefined equations achieved maximum relative errors of 3.1% for *E_0_*, 1.3% for *τ* and 6.0% for *E_1_* across the investigated parameter space. Detailed error analyses (Figure 3C–F) illustrate the accuracy of these equations for microgel radii ranging from 20 µm to 50 µm, maximum contact forces from 2 nN to 10 nN, and fully relaxed Young's moduli up to 2000 Pa. Consistent with experimental observations, the relaxation modulus (*E_1_*) was considered up to 20% of the fully relaxed Young's modulus (*E_0_*). Specifically, Figure 3C shows the relative error in calculated*E_0_* (*E_0,cal_*) across varying *F_1_* and *R*, with the absolute error peaking at *E_0_* = 2000 Pa and *F_1_* = 2 nN. Similarly, the relative error for the relaxation time constant τ remained consistently low (1.3%, Figure 3D). The absolute error in calculating *E_1_* was most pronounced at fully relaxed Young's Moduli (*E_0_*) of 500 Pa and 2000 Pa, particularly when the difference between *F_1_* and *F_0_* was minimal, corresponding to the lowest viscosity condition. Figure 3E and F illustrate these absolute errors in the relaxation modulus (*E_1_*) at *E_0_* = 500 Pa and *E_0_* = 2000 Pa, respectively, at a cantilever velocity *v* = 3 µm s^−1^ and *τ* = 0.5 s, varying *F_1_* and microgel radii (*R*). Comparable absolute errors for *E_1_* were observed at intermediate fully relaxed Young's moduli (*E_0_* = 1000 Pa and 1500 Pa).

Notably, at very low cantilever velocities (i.e., 1 µm s^−1^), the apparent viscosity decreases significantly, causing the predefined equations for *E_1_* to exhibit higher relative errors (up to 30%). Additionally, the equations were specifically optimized for a spherical AFM probe radius of 5 µm. For significantly smaller microgels (<20 µm) or different cantilever geometries, re‐optimization of the coefficients α, β, γ may be necessary. For users wishing to apply this framework to different probe geometries or materials outside our tested range, these coefficients can be readily re‐optimized. The process involves (1) using the FE model to generate a new synthetic dataset for the desired parameter space, and (2) employing the same optimization procedure described in our methods to fit new coefficients to the analytical equations. While the specific coefficients are application‐dependent, the overall analytical framework and the workflow for their determination are broadly applicable, providing a robust template for rapid viscoelastic characterization in diverse experimental contexts. Within the validated parameter ranges, however, these equations provide a rapid, accurate, and straightforward method for extracting viscoelastic material parameters from AFM‐based stress relaxation experiments, significantly enhancing evaluation efficiency without significant compromises in accuracy.

## 3 Conclusion

This study introduces an integrated approach for characterizing scale‐dependent viscoelastic properties in hydrogels, combining advanced microfluidic fabrication, AFM‐based nanoindentation, and computational modeling.

We employed a modular hydrogel system composed of two IPNs, enabling precise and independent control over elasticity and stress relaxation. For the first time, monodisperse microgels were fabricated from this IPN system using microfluidic techniques with integrated electrodes, enabling rapid and controlled mixing of hydrogel precursors via emulsion droplet coalescence.^[^ ^55^ ^]^ Bulk gels prepared from identical formulations provided a direct basis for comparing viscoelastic properties across different length scales. AFM nanoindentation experiments, supported by FE simulations, revealed that hydrogel mechanics cannot be extrapolated across length scales, highlighting the necessity of scale‐specific characterization for accurate biomaterial design.

To facilitate practical implementation, we developed a universal analytical framework that rapidly extracts key viscoelastic parameters – fully relaxed elastic modulus (*E_0_*), relaxation modulus (*E_1_*), and relaxation time (*τ*) – from AFM stress relaxation data. This approach achieves remarkable accuracy (maximum errors: 3.1% for *E_0_*, 1.3% for *τ*, 6.0% for *E_1_*) across physiologically relevant ranges while eliminating the need for computationally intensive optimization.

This integrated experimental‐computational strategy provides a robust platform for designing viscoelastic hydrogels with tailored properties and is particularly valuable for applications in tissue engineering and cellular mechanobiology, where precise control over time‐dependent mechanical properties is crucial for guiding cell behavior.

## 4 Experimental Section

### Hydrogel Components

The IPN hydrogels were prepared from starPEG and heparin precursors. The starPEG components included a commercially sourced starPEG‐Thiol (0.83 mM; MW 10,600 g mol^−1^; Polymer Source, Inc.) and an in‐house synthesized starPEG‐(KA)_7_ (0.28 mM; MW 17,000 g mol^−1^). The heparin components comprised non‐functionalized heparin (0.28 mM; MW 14,000 g mol^−1^; Merck KGaA) and an in‐house synthesized maleimide‐functionalized heparin HM6 (0.83 mM; MW 15,000 g mol^−1^). The viscoelastic properties of the IPN hydrogels were precisely tuned by controlling the network ratio, with the physical (non‐covalent) network constituting 25% of the total molar composition and the covalent network comprising the remaining 75%.

### Microfluidic Device Fabrication and Setup Design

Water‐in‐oil emulsions were generated using custom polydimethylsiloxane (PDMS) microfluidic devices fabricated through standard photolithography and soft lithography techniques.^[^ ^55^ ^]^ The microfluidic channels were uniformly designed with a width of 50 µm for the dispersed phases, continuous phase, and droplet‐transporting channel. The fabrication process began by spin‐coating a 3‐inch silicon wafer (Siegert Wafer) with SU‐8 2025 photoresist (Micro Resist Technology) to create a master mold. Channel patterns were defined using a mask aligner (MJB3, Suess MicroTec) and a printed film mask. After exposure and baking, the uncured photoresist was removed using a developer (mr‐Dev 600, Micro Resist Technology). The microchannel structure was then replicated via PDMS replica molding. A mixture of PDMS base and curing agent (Sylgard 184, Dow Corning) at a 10:1 ratio was degassed using a planetary centrifugal mixer (ARE‐250, Thinky), poured over the master mold and cured at 65 °C for 2 hours. After demolding, 1.0 mm inlet/outlet ports were punched with a biopsy tool (KAI Medical), and the PDMS channel side was bonded to a glass microscopy slide via oxygen plasma treatment (80 W for 15 s, MiniFlecto 10, Plasma Technology). To enable electric field‐assisted droplet manipulation, electrode microchannels were filled with a low‐melting metal alloy (51% In, 32.5% Bi, 16.5% Sn, Indium Corporation of America) to generate localized strong electric fields. Non‐electrode microchannels were hydrophobized by injecting a 1% (v/v) solution of (tridecafluoro‐1,1,2,2‐tetrahydrooctyl)trichlorosilane (Gelest) in Novec 7500 (IoLiTec). During operation, dispersed phases were delivered through 500 µL gastight syringes (Hamilton) and continuous phases via 3 mL disposable syringes (BD Luer‐lock), all controlled by high‐precision syringe pumps (Pico Plus Elite) connected through PE tubing (0.38 mm ID). The emulsion process was monitored in real‐time using an inverted bright‐field microscope (Axio Vert.A1, Carl Zeiss) equipped with a high‐speed digital camera (Miro C110, Vision Research Inc.).

### Microgel Fabrication

Microgel fabrication was initiated by injecting the first dispersed phase (DP 1), containing 0.83 mM starPEG‐Thiol and 0.28 mM starPEG‐(KA)_7_, at a flow rate of 50 µL hr^−1^ (Figure 1B). Emulsification was achieved using two continuous phases of 2% (w/w) PFPE‐PEG‐PFPE triblock copolymer surfactant (RAN Biotechnologies) in Novec 7500, delivered at 500 µL hr^−1^. The second dispersed phase (DP 2), containing 0.83 mM heparin‐HM6 and 0.28 mM heparin, was introduced through a side channel at matching flow rates, creating paired DP 1/DP 2 droplets. These pairs coalesced into single droplets upon exposure to a 50 kV electric field at integrated electrodes. Subsequent rapid mixing and gelation occurred in a meandering channel, driven by the chemical reaction between the components of DP 1 and DP 2. Microgels were collected every 10 minutes and purified through three washes with 20% (v/v) 1H,1H,2H,2H‐perfluoro‐1‐octanol (PFO, Sigma‐Aldrich) in Novec 7500, before being transferred to PBS buffer.

### Bulk Gel Preparation

Preparation of IPN bulk gels involved two separate precursor solutions: (1) a starPEG solution prepared by combining starPEG‐(KA)_7_ with starPEG‐Thiol, and (2) a heparin solution prepared by combining non‐functionalized heparin with heparin HM6. These solutions were mixed at a 25:75 molar ratio of non‐covalent to covalent networks – matching the microgel formulation – and deposited as droplets (20 µL) in Petri dishes filled with PBS. After crosslinking, the resulting hydrogel droplets measured approximately 5 mm in diameter and 1 mm in height.

### Mechanical Characterization of Microgels and Bulk Gels

Mechanical properties were quantified using a NanoWizard 4 AFM (Bruker) coupled with an inverted optical microscope (Observer A1, Zeiss). A tipless cantilever (PNP‐TR‐TL, NanoWorld; k = 0.08 N/m) fitted with a 10 µm colloidal force probe (Microparticles GmbH) was employed for both indentation and stress relaxation measurements. The cantilever was calibrated before each experiment using the thermal noise method^[^ ^59^, ^60^ ^]^ with all measurements conducted in PBS at room temperature.

For indentation testing, the colloidal probe was positioned over the center of individual microgels or bulk gels. Force‐distance curves were recorded at three approach/retract velocities (1, 3, and 5 µm s^−1^  ). A constant contact force of 2 nN was maintained, resulting in typical indentation depths of approximately 1 µm. The Young's modulus was determined by fitting the approach segment of the force–distance curve to the Hertz model for a spherical indenter. Microgels analysis incorporated a double‐contact correction to account for additional deformation from the counter pressure at the microgel's underside.^[^ ^61^, ^62^, ^63^ ^]^ All data processing was performed using the AFM's native software with custom analysis algorithms.

Stress relaxation measurements followed a three‐step protocol (Figure 2B). In the indentation phase (phase 1), the cantilever advanced at controlled velocities (1, 3, or 5 µm s^−1^) until reaching a 10 nN loading force. Since the expected drag forces were in the piconewton range ‐ approximately four orders of magnitude smaller than the target indentation forces (10 nN) and the measured relaxation forces ‐ it was concluded that the contribution of hydrodynamic drag was negligible and did not affect the accuracy of the extracted viscoelastic parameters. The subsequent hold phase (phase 2) maintained constant displacement for 10 seconds while monitoring force decay, allowing characterization of time‐dependent viscoelastic behavior. Finally, in the retraction phase (phase 3), the cantilever was withdrawn from the sample surface.

### Determination of FE Models

To computationally replicate the AFM experiments, FE models were developed representing both microgel and bulk gel geometries. Microgels were modelled as spheres (Figure S1A, Supporting Information), while bulk gels were approximated as cylinders with 1000 µm height to match experimental conditions (Figure S1B, Supporting Information). Both geometries incorporated rotational symmetry to optimize computational efficiency.

A mesh convergence study established the optimal element density for accurate parameter determination (Figure S1C, Supporting Information). The microgel model comprised 23,690 elements (24,130 degrees of freedom), while the bulk gel model used 14,714 elements (15,080 degrees of freedom). Triangular elements with quadratic shape functions were employed throughout, with localized mesh refinement in the bulk gel's contact region to resolve steep stress gradients. The AFM indenter was modeled as a rigid 10 µm sphere with frictionless contact assumptions.

### Finite Element Simulation‐Based Analysis of Viscoelastic Material Properties

FE simulations were employed to analyze the viscoelastic behavior of microgels and bulk gels, coupled with an optimization procedure to determine material parameters that best fit experimental data. Given the significant displacements observed during AFM indentation, the material model was based on finite strain theory. The viscoelastic material behavior was described using a combination of a Neo‐Hookean model and a single‐term Prony series. The Neo‐Hookean model captured the nonlinear elastic response at large deformations, while the Prony series component accounted for the time‐dependent viscous response. This approach was equivalent to a generalized Maxwell model with a single spring‐dashpot element, commonly used in relaxation experiments.^[^ ^64^, ^65^, ^66^ ^]^

The time‐dependent Young's modulus *E(t)* was expressed in one dimension as:

(4) $$E\left(t\right)=E_{0}+E_{1}e^{-\left(t/\tau \right)}\;$$

where *E*_0_ was the fully relaxed Young's modulus, *E*_1_ represents the initial viscous contribution, and τ was the relaxation time constant. The instantaneous (unrelaxed) modulus was given by *E_u_* =  *E*_0_ + *E*_1_, and τ can be interpreted as τ =  η/*E*_1_, with η being the dashpot viscosity. To account for the nearly incompressible nature of the hydrogels, the Poisson's ratio was set to ν = 0.45.^[^ ^14^, ^62^ ^]^

Parameter optimization proceeded in two sequential phases:

- **Relaxed Modulus (*E_0_*)**: The fully relaxed behavior was determined using phase 2 of the experiment, during which the indenter was held at the maximum displacement (*u_max_*) for 10 seconds (Figure 2B).
- **Viscous Parameters (*E_1_* and *τ*)**: These parameters were optimized using the stress relaxation data from phase 2, where the displacement remained constant, and the stress decay was governed solely by viscous behavior.

This two‐step approach minimized inaccuracies caused by potential nonlinear elastic effects not fully captured by the Neo‐Hookean model during the initial loading phase.

### FE Simulation and Parameter Optimization

FE simulations were performed in Abaqus (Dassault Systèmes. Abaqus 2024: Unified FEA Software), a commercial finite element software, with optimization implemented in Python (Python Software Foundation (2023)) using the “mystic” library, which combines evolutionary strategies with gradient‐based methods.

### Elastic Modulus (*E_0_*) Optimization

*E_0_* was optimized using the maximum indenter displacement (*u_max_*) from the experiments as the boundary condition. Since experiments were conducted at three indenter velocities (*v_1_* = 1 µm s^−1^, *v_2_* = 3 µm s^−1^, and *v_3_* = 5 µm s^−1^), three separate simulations were executed for each candidate *E_0_* value. In these simulations, the material was modeled using a Neo‐Hookean model (without viscosity). The simulated contact force (*F_sim_)* at the end of the relaxation phase (phase 2) was compared with the corresponding experimental force (*F_exp_)*. The first objective function *z*_1_ of the optimization was defined as:

(5) $$z_{1}=\sum_{i=1}^{3}\sqrt{\left(F_{\mathrm{sim},i}-F_{\exp,i}\right)}$$

where *F*_sim,*i*_ and *F*_exp, *i*_ were the contact forces at the end of phase 2 for the three velocities (*v*_1_, *v*_2_ and *v*_3_).

### Optimizing the Viscous Parameters (*E_1_* and *τ*)

The viscous parameters (*E_1_* and τ) were optimized by capturing the full force–time response during phases 1 and 2. In these simulations, the experimental displacement–time profile was imposed as the boundary condition. The simulated force–time curves (*F_sim_)* were compared with the experimental curves (*F_exp_)* at all time steps across the three velocities. The second objective function (z_2_) was defined as:

(6) $$z_{2}=\sum_{i=1}^{3}\sqrt{\sum_{j=1}^{n_{i}}{\left(\frac{\left(F_{\mathrm{sim},ij}-F_{\exp,ij}\right)t_{j}}{t_{\mathrm{ges}}}\right)}^{2}}$$

where *i* corresponds to the different velocities (*v_i_*) and *n_i_* was the total number of time steps of the simulation in phase 2. Consequently, *F*_sim,*i* *j*_ were the contact forces at the corresponding time step at the time *t_j_* and *F*_exp, *i* *j*_ was the contact force in the experiments, which was linearly interpolated from the experimental data for the time *t_j_*. The factor (*t_j_*/*t*_ges_) with *t*_ges_ = 10 s was used as weighting factor for a more reliable optimization result.

### Optimization of Material Parameter Equations

A mathematical model was constructed to estimate *E_0_, E_1_* and *τ* (Figure 3A) and the coefficients in these material parameter equations were optimized over the data range specified in Figures S2–S5 (Supporting Information). To achieve this, 1,920 simulations replicating the AFM mechanical experiments were performed to fine‐tune the coefficients α_1_ α_2_, α_3_, β_1_, γ_1_, γ_2_, γ_3_. Each simulation tested a unique combination of the following variables:

- Elastic modulus (*E_0_*): 500, 1000, 1500, 2000 Pa
- Microgel radius (*R*): 20, 35, 50 µm
- Maximum contact force (*F_1_*): 2, 4, 6, 8, 10 nN
- Ratio *E_1_/E_0_*: 0.05, 0.1, 0.15, 0.2
- Relaxation time constant (*τ*): 0.05, 0.1, 0.15, 0.2 s
- Indenter velocity (*v*): 3 µm s^−1^, 5 µm s^−1^.

The radius of the indenter cantilever was fixed at 5 µm.

From each simulation, the values of *F_0_*, *F_1_*, *t_1_*, *t_mid_* and *u_max_* were exported and used as input data for the non‐linear regression analysis. The predefined regression equations were initially derived from mechanical assumptions. The coefficients of these equations were optimized by applying a combination of evolutionary strategies and gradient‐based methods implemented in the “mystic” Python package. The optimization error was evaluated using the least‐squares criterion:

(7) $$E_{o}=\frac{1}{1920}\sum_{i=1}^{1920}{\left(ô-o\right)}^{2}$$

where *E_o_* denotes the error of the predefined equation predicting the parameter *o*, *o* was the value of the output variable (*E_0_, E_1_* or *τ)*, and ô was the corresponding prediction obtained from the predefined equation. Minimization of *E_o_* yields to the optimal values of the model coefficients. The predefined equations were refined iteratively through a trial‐and‐error process, guided by the observed correlations between input and output variables. The best fitting results were obtained by sequentially optimizing the output variables, starting with *E_o_*, followed by *τ*, and finally *E_1_*.

The prediction of the purely elastic modulus (*E_0_*) was based on the relaxed contact force (*F_0_*), the corresponding indentation (*u*_max_) and the microgel radius (*R*). Since only viscoelastic materials were considered, *F_0_* could not be predicted but depended on *F_1_, E_1_, τ* and *v*. Figure 3C presents an error plot comparing the predictions of the predefined equation with the simulated output values for the conditions *E_1_/E_0_* = 0.05, *τ* = 0.5 s, and *v* = 3 µm s^−1^, representing the lowest viscosity and the largest absolute errors for *E_0_*. Similarly, Figure 3D illustrates an error plot showing the maximal absolute errors for *τ* under the conditions *v* = 5 µm s^−1^, *E_0_* = 2000 Pa, and *E_1_* = 400 Pa. For *E_1_*, the maximal absolute errors were observed in two distinct cases with *E_0_* = 500 Pa (Figure 3E) and *E_0_* = 2000 Pa (Figure 3F), both at *v* = 5 µm s^−1^. The corresponding qualitative values of all error plots were provided in Figures S2–S5 (Supporting Information).

## Conflict of Interest

The authors declare no conflict of interest.

## Supporting information



Supporting Information

## Data Availability

The data that support the findings of this study are available in the supplementary material of this article.
