# Supplementary material for: Scale‐Specific Viscoelastic Characterization of Hydrogels: Integrated AFM and Finite Element Modeling
Source: Small. 2025 Dec 3;22(15):e07835. doi: 10.1002/smll.202507835 (PMC12980481; doi:10.1002/smll.202507835)
Supplement: Supplementary file 1 — Supporting Information [file SMLL-22-e07835-s001.docx]

Supporting Information

**Scale-Specific Viscoelastic Characterization of Hydrogels:
Integrated AFM and Finite Element Modeling**

*Nicole Fertala^*^, Klemens Uhlmann, Evgeny Grigoryev, Prannoy Seth, Jens Friedrichs, Julian Thiele, Carsten Werner, Daniel Balzani*


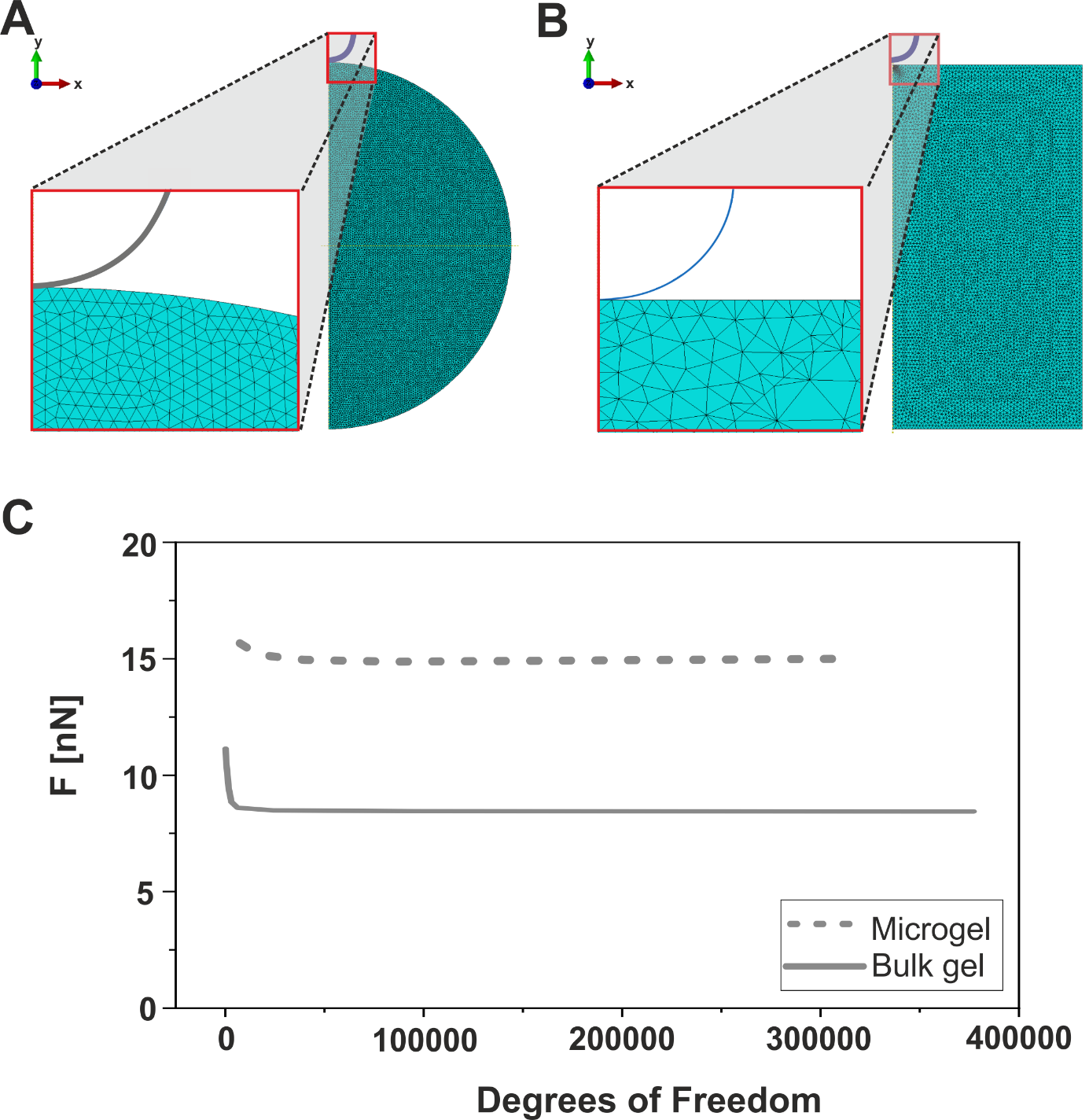


**Figure S1: FE Model Geometry and Mesh Convergence Analysis.** FE model geometry and mesh used for nanoindentation simulations of microgels **(A)** and bulk gels **(B)**. Insets (red boxes) in both panels provide enlarged views of the contact region between the spherical AFM probe and the sample surface, highlighting local mesh refinement. **(C)** Mesh convergence analysis showing the relationship between computed contact force and increasing degrees of freedom for microgels (dashed line) and bulk gels (solid line), demonstrating that further mesh refinement yields negligible changes in the simulated contact force, thus confirming convergence.

| fully relaxed Young´s modulus = 500 Pa | | | | | |
| --- | --- | --- | --- | --- | --- |
| R = 20 µm | | R = 35 µm | | R = 50 µm | |
| F_1_ in nN | E_0_ in Pa | F_1_ in nN | E_0_ in Pa | F_1_ in nN | E_0_ in Pa |
| 2 | 487.64 | 2 | 495.22 | 2 | 499.41 |
| 4 | 494.67 | 4 | 493.01 | 4 | 492.86 |
| 6 | 501.75 | 6 | 492.26 | 6 | 489.97 |
| 8 | 508.72 | 8 | 491.45 | 8 | 487.10 |
| 10 | 515.67 | 10 | 491.46 | 10 | 484.34 |
| Elastic modulus = 1000 Pa | | | | | |
| R = 20 µm | | R = 35 µm | | R = 50 µm | |
| F_1_ in nN | E_0_ in Pa | F_1_ in nN | E_0_ in Pa | F_1_ in nN | E_0_ in Pa |
| 2 | 970.78 | 2 | 995.35 | 2 | 1013.84 |
| 4 | 975.29 | 4 | 990.44 | 4 | 998.82 |
| 6 | 982.46 | 6 | 988.51 | 6 | 990.70 |
| 8 | 989.34 | 8 | 986.01 | 8 | 985.72 |
| 10 | 996.32 | 10 | 984.73 | 10 | 982.37 |
| Elastic modulus = 1500 Pa | | | | | |
| R = 20 µm | | R = 35 µm | | R = 50 µm | |
| F_1_ in nN | E_0_ in Pa | F_1_ in nN | E_0_ in Pa | F_1_ in nN | E_0_ in Pa |
| 2 | 1455.52 | 2 | 1503.36 | 2 | 1521.30 |
| 4 | 1457.24 | 4 | 1492.06 | 4 | 1511.89 |
| 6 | 1462.93 | 6 | 1485.65 | 6 | 1498.23 |
| 8 | 1469.84 | 8 | 1482.91 | 8 | 1487.79 |
| 10 | 1476.99 | 10 | 1478.85 | 10 | 1485.71 |
| Elastic modulus = 2000 Pa | | | | | |
| R = 20 µm | | R = 35 µm | | R = 50 µm | |
| F_1_ in nN | E_0_ in Pa | F_1_ in nN | E_0_ in Pa | F_1_ in nN | E_0_ in Pa |
| 2 | 1942.10 | 2 | 2019.59 | 2 | 2058.18 |
| 4 | 1941.57 | 4 | 1990.65 | 4 | 2027.68 |
| 6 | 1945.68 | 6 | 1986.45 | 6 | 2012.78 |
| 8 | 1950.58 | 8 | 1980.87 | 8 | 1997.64 |
| 10 | 1958.36 | 10 | 1976.67 | 10 | 1989.52 |

**Figure S2:** Estimated values of the fully relaxed Young's modulus (*E₀*) compared to actual values, analyzed as a function of the predefined maximum contact force (*F_1_*) and microgel radius (*R*).

| Tau = 0.5 | | | | | |
| --- | --- | --- | --- | --- | --- |
| R = 20 µm | | R = 35 µm | | R = 50 µm | |
| F_1_ in nN | tau | F_1_ in nN | tau | F_1_ in nN | tau |
| 2 | 0.50628 | 2 | 0.50645 | 2 | 0.50657 |
| 4 | 0.50593 | 4 | 0.50622 | 4 | 0.50636 |
| 6 | 0.50571 | 6 | 0.50603 | 6 | 0.50592 |
| 8 | 0.50544 | 8 | 0.50569 | 8 | 0.50593 |
| 10 | 0.50531 | 10 | 0.50557 | 10 | 0.50572 |
| Tau = 1.0 | | | | | |
| R = 20 µm | | R = 35 µm | | R = 50 µm | |
| F_1_ in nN | tau | F_1_ in nN | tau | F_1_ in nN | tau |
| 2 | 1.01254 | 2 | 1.01071 | 2 | 1.01304 |
| 4 | 1.01192 | 4 | 1.01235 | 4 | 1.01262 |
| 6 | 1.01137 | 6 | 1.01201 | 6 | 1.01196 |
| 8 | 1.01081 | 8 | 1.01124 | 8 | 1.01192 |
| 10 | 1.01045 | 10 | 1.01097 | 10 | 1.01127 |
| Tau = 1.5 | | | | | |
| R = 20 µm | | R = 35 µm | | R = 50 µm | |
| F_1_ in nN | tau | F_1_ in nN | tau | F_1_ in nN | tau |
| 2 | 1.51627 | 2 | 1.51328 | 2 | 1.51704 |
| 4 | 1.51546 | 4 | 1.51615 | 4 | 1.51622 |
| 6 | 1.51389 | 6 | 1.51549 | 6 | 1.51556 |
| 8 | 1.51326 | 8 | 1.51433 | 8 | 1.51469 |
| 10 | 1.51275 | 10 | 1.51366 | 10 | 1.51413 |
| Tau = 2.0 | | | | | |
| R = 20 µm | | R = 35 µm | | R = 50 µm | |
| F_1_ in nN | tau | F_1_ in nN | tau | F_1_ in nN | tau |
| 2 | 2.00582 | 2 | 2.00585 | 2 | 2.00670 |
| 4 | 2.00582 | 4 | 2.00671 | 4 | 2.00684 |
| 6 | 2.00154 | 6 | 2.00676 | 6 | 2.00633 |
| 8 | 2.00056 | 8 | 2.00189 | 8 | 2.00224 |
| 10 | 1.99956 | 10 | 2.00100 | 10 | 2.00147 |

**Figure S3:** Estimated values of the relaxation time (*τ*) compared to actual values, analyzed as a function of the predefined maximum contact force (*F_1_*) and the radius (*R*) of the microgel.

| Relaxation modulus = 25 Pa | | | | | |
| --- | --- | --- | --- | --- | --- |
| R = 20 µm | | R = 35 µm | | R = 50 µm | |
| F_1_ in nN | E_1_ in Pa | F_1_ in nN | E_1_ in Pa | F_1_ in nN | E_1_ in Pa |
| 2 | 24.581 | 2 | 24.426 | 2 | 24.407 |
| 4 | 24.473 | 4 | 24.017 | 4 | 23.908 |
| 6 | 24.839 | 6 | 24.121 | 6 | 23.847 |
| 8 | 25.519 | 8 | 24.454 | 8 | 24.072 |
| 10 | 26.361 | 10 | 25.014 | 10 | 24.459 |
| Relaxation modulus = 50 Pa | | | | | |
| R = 20 µm | | R = 35 µm | | R = 50 µm | |
| F_1_ in nN | E_1_ in Pa | F_1_ in nN | E_1_ in Pa | F_1_ in nN | E_1_ in Pa |
| 2 | 48.152 | 2 | 47.895 | 2 | 47.832 |
| 4 | 48.174 | 4 | 47.361 | 4 | 47.081 |
| 6 | 49.086 | 6 | 47.661 | 6 | 47.158 |
| 8 | 50.472 | 8 | 48.357 | 8 | 47.688 |
| 10 | 52.191 | 10 | 49.466 | 10 | 48.475 |
| Relaxation modulus = 75 Pa | | | | | |
| R = 20 µm | | R = 35 µm | | R = 50 µm | |
| F_1_ in nN | E_1_ in Pa | F_1_ in nN | E_1_ in Pa | F_1_ in nN | E_1_ in Pa |
| 2 | 71.903 | 2 | 71.361 | 2 | 71.344 |
| 4 | 72.320 | 4 | 71.065 | 4 | 70.477 |
| 6 | 73.825 | 6 | 71.763 | 6 | 70.925 |
| 8 | 75.974 | 8 | 72.958 | 8 | 71.876 |
| 10 | 78.673 | 10 | 74.674 | 10 | 73.209 |
| Relaxation modulus = 100 Pa | | | | | |
| R = 20 µm | | R = 35 µm | | R = 50 µm | |
| F_1_ in nN | E_1_ in Pa | F_1_ in nN | E_1_ in Pa | F_1_ in nN | E_1_ in Pa |
| 2 | 96.089 | 2 | 95.366 | 2 | 94.796 |
| 4 | 97.011 | 4 | 95.472 | 4 | 94.693 |
| 6 | 99.224 | 6 | 96.595 | 6 | 95.558 |
| 8 | 102.362 | 8 | 98.509 | 8 | 97.052 |
| 10 | 106.030 | 10 | 100.979 | 10 | 98.879 |

**Figure S4:** Estimated values for the relaxation modulus (*E₁*) at the lower boundary (with *E₀* = 500 Pa) compared to actual values, analyzed as a function of the predefined maximum contact force (*F_1_*) and the radius (*R*) of the microgel.

| Relaxation modulus = 100 Pa | | | | | |
| --- | --- | --- | --- | --- | --- |
| R = 20 µm | | R = 35 µm | | R = 50 µm | |
| F_1_ in nN | E_1_ in Pa | F_1_ in nN | E_1_ in Pa | F_1_ in nN | E_1_ in Pa |
| 2 | 101.846 | 2 | 96.8616 | 2 | 101.809 |
| 4 | 100.060 | 4 | 96.8616 | 4 | 99.651 |
| 6 | 99.015 | 6 | 96.8616 | 6 | 98.332 |
| 8 | 98.312 | 8 | 96.8616 | 8 | 97.630 |
| 10 | 97.875 | 10 | 96.8616 | 10 | 96.683 |
| Relaxation modulus = 200 Pa | | | | | |
| R = 20 µm | | R = 35 µm | | R = 50 µm | |
| F_1_ in nN | E_1_ in Pa | F_1_ in nN | E_1_ in Pa | F_1_ in nN | E_1_ in Pa |
| 2 | 197.908 | 2 | 198.019 | 2 | 198.029 |
| 4 | 195.455 | 4 | 194.523 | 4 | 194.558 |
| 6 | 193.773 | 6 | 192.965 | 6 | 192.512 |
| 8 | 192.613 | 8 | 191.578 | 8 | 191.324 |
| 10 | 192.321 | 10 | 190.113 | 10 | 189.870 |
| Relaxation modulus = 300 Pa | | | | | |
| R = 20 µm | | R = 35 µm | | R = 50 µm | |
| F_1_ in nN | E_1_ in Pa | F_1_ in nN | E_1_ in Pa | F_1_ in nN | E_1_ in Pa |
| 2 | 293.290 | 2 | 293.237 | 2 | 292.713 |
| 4 | 290.422 | 4 | 288.993 | 4 | 289.209 |
| 6 | 288.803 | 6 | 286.562 | 6 | 286.938 |
| 8 | 287.617 | 8 | 285.443 | 8 | 285.376 |
| 10 | 287.124 | 10 | 284.614 | 10 | 283.782 |
| Relaxation modulus = 400 Pa | | | | | |
| R = 20 µm | | R = 35 µm | | R = 50 µm | |
| F_1_ in nN | E_1_ in Pa | F_1_ in nN | E_1_ in Pa | F_1_ in nN | E_1_ in Pa |
| 2 | 388.640 | 2 | 387.685 | 2 | 387.658 |
| 4 | 385.592 | 4 | 384.285 | 4 | 383.811 |
| 6 | 384.446 | 6 | 382.171 | 6 | 382.422 |
| 8 | 384.358 | 8 | 381.458 | 8 | 379.188 |
| 10 | 384.400 | 10 | 380.968 | 10 | 379.736 |

**Figure S5:** Estimated values for the relaxation modulus (*E₁*) at the upper boundary (with *E₀* = 2000 Pa) compared to actual values, analyzed as a function of the predefined maximum contact force (*F_1_*) and the radius (*R*) of the microgel.
